# Evaluation of the objective structured clinical examination in the assessment of nuclear medicine residency training

**DOI:** 10.1186/s13244-026-02340-2

**Published:** 2026-07-01

**Authors:** Lijuan Di, Jianhua Zhang, Guangyu Zhao, Yan Fan

**Affiliations:** Department of Nuclear Medicine, Peking University First Hospital, Beijing, China

**Keywords:** Objective structured clinical examination, Evaluation, Resident training, Nuclear medicine, Clinical competence

## Abstract

**Objectives:**

We evaluated the capacity of the Objective Structured Clinical Examination (OSCE) to certify resident competency in nuclear medicine (NM).

**Materials and methods:**

A retrospective analysis was conducted on the OSCE scores of residents who participated in the examination for the certification of NM physicians in Beijing from 2019 to 2024, with a focus on difficulty, discrimination, reliability, and validity.

**Results:**

The OSCE is comprised of six stations, including high-radioactivity room operations, imaging room operations, PET/CT case analysis, SPECT/CT case analysis, patient interviews and report writing. The mean total OSCE scores (mean ± SD) were 88.58 ± 2.37 points (2019), 89.13 ± 2.72 points (2020), 86.15 ± 2.75 points (2021), 92.96 ± 2.31 points (2022), 91.11 ± 1.37 points (2023), and 89.08 ± 2.75 points (2024), respectively. The overall difficulty coefficients of the OSCE were 0.89, 0.89, 0.86, 0.88, 0.91, and 0.89, respectively. The internal consistency reliability values (Cronbach’s α) were 0.66, 0.76, 0.57, 0.28, 0.05, and 0.69, respectively, across the years. The station-level discrimination indices ranged from 0.46 to 0.83. Among the 30 stations that were double-scored by two examiners, 73.33% (22/30) of the stations demonstrated excellent interrater consistency.

**Conclusion:**

The OSCE effectively simulates clinical environments and provides a comprehensive assessment of the clinical competence of NM residents.

**Critical relevance statement:**

This study conducted a multidimensional evaluation of 6-year OSCE data, focusing on difficulty, discrimination, reliability, and validity, demonstrating its effectiveness in simulating clinical environments and assessing the clinical competence of NM residents.

**Key Points:**

- Compared to the numerous studies on medical students, relatively few studies on the OSCE target NM residents.
- The OSCE exhibited acceptable station difficulty and discrimination, moderate reliability, and satisfactory content validity for assessing NM residents.

**Graphical Abstract:**

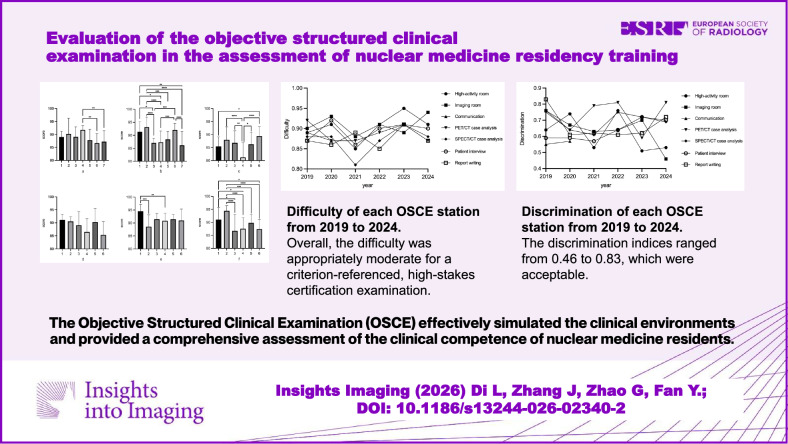

## Introduction

The Objective Structured Clinical Examination (OSCE) was first proposed by Harden in 1975 as a tool for assessing the comprehensive competence of clinical physicians [1]. By simulating various clinical scenarios, the OSCE allows for the evaluation of the professional knowledge, clinical skills, clinical reasoning, communication, and humanistic quality abilities of the candidates [1, 2]. As an indispensable complement to traditional written examinations, the OSCE can be used to assess the competence of health professionals at different levels, such as undergraduate medical students and postgraduate residents [3–6]. Although the scholarly discourse on OSCE design and validation has been predominantly demonstrated by North American and European investigators, contributions from Asian countries remain scarce [7]. Furthermore, most OSCE research has focused on medical students, with relatively few studies targeting resident physicians [8].

In Beijing, the successful completion of the OSCE is a mandatory gateway to standardized residency training (SRT) certification, which is a prerequisite for taking the attending-physician licensing examination and (under the national “integrated education and training” pathway) for the conferral of postgraduate degrees. The final summative assessment in NM is supervised by the Science and Education Department of the Beijing Municipal Health Commission, which is coordinated by the Beijing Health Human Resources Development Center and the Beijing Association for Medical Education and implemented by the Beijing Board of Nuclear Medicine Residency at a designated assessment centre. Since 2014, the OSCE has served as the capstone evaluation for NM residents in Beijing. The OSCE scheme is designed by the Beijing Board of Nuclear Medicine Residency. Moreover, the Department of Nuclear Medicine at Peking University First Hospital (which functions as the leading institution and assessment centre) has accumulated extensive experience in the administration of the OSCE.

This study aimed to conduct a comprehensive analysis of OSCE data from 2019 to 2024, in order to accurately evaluate the validity and reliability of the OSCE. The findings are contextualised within prevailing training challenges, and concrete recommendations are offered to refine future iterations of the SRT programme.

## Methods

### OSCE

The OSCE scores of NM residents who completed three years of SRT in Beijing and also completed the final certifying examination between 2019 and 2024 were analysed [9]. In accordance with the Detailed Rules for Beijing SRT, the summative assessment was designed to evaluate residents’ clinical competence across Canadian Medical Education Directions for Specialists (CanMEDS)-aligned domains, including patient consultation, clinical reasoning, operation skills, diagnostic test utilisation, medical documentation, and communication [10]. The Beijing Board of Nuclear Medicine Residency has translated these competencies into a six-station OSCE blueprint that operationalises the CanMEDS framework within the nuclear-medicine context.

1. High-radioactivity room station
  Core tasks: radiopharmaceutical handling, shielding, dose calibration, and radioactive waste disposal.
2. Imaging room station
  Core tasks: protocol selection, acquisition parameters, image acquisition and postprocessing.
3. Patient interview/communication station
  Core tasks: collection of information regarding patient history and physician-patient communication using standardised patients (SPs).
4. Positron emission tomograph (PET)/computed tomography (CT) Case-Analysis Station
  Core tasks: integration of clinical data and PET/CT images to formulate a differential diagnosis.
5. Single photon emission computed tomography (SPECT)/CT case-analysis station
  Core tasks: integration of clinical data and SPECT/CT images to formulate a differential diagnosis.
6. Report writing station

Core tasks: generation of a concise, structured diagnostic report from provided images and clinical data.

Stations 1–5 were independently scored by two board-certified examiners (ranks of associate chief physician or higher); moreover, the mean of the two scores constituted the station mark. Due to staffing shortages, the Report Writing Station was assessed by a single examiner. SPs received standardised training 1–2 days before the examination, and all of the examiners participated in two training sessions to maximise interrater consistency. The pass standard was defined as a total score ≥ 80 points, with a minimum score of 80 points for at least five of the six stations being utilised. The content, duration, and weight for each station are summarised in Table 1.

**Table 1:** OSCE schemes

Year	Station		Weight coefficient	Exam duration (min)
2019～2020	SP station	Patient interview	15%	18
		Communication	10%	18
	Non-SP station	High-activity room	10%	18
		Imaging room	15%	18
		Case analysis	25%	18
		Report writing	25%	30
		Total	100%	120
2021～2024	SP station^a^	Patient interview	15%	18
	Non-SP station	High-activity room	10%	18
		Imaging room	15%	18
		SPECT/CT case analysis	20%	18
		PET/CT case analysis	20%	18
		Report writing	20%	40
		Total	100%	130

*SP* standardised patient

^a^ No SP was included in 2020 for the COVID-19 pandemic

### Objectives

The OSCE scores from 2019 to 2024 were entered into the database. According to the Classical Test Theory, we examined (i) station-level difficulty and discrimination, (ii) overall test difficulty, (iii) internal consistency reliability, (iv) interrater reliability, (5) construct validity, and (vi) content validity. Difficulty (P) was quantified as *P* = *X*/*K*, where *X* denotes the mean score, and *K* denotes the full score; additionally, higher *P* values indicate an easier examination. Discrimination (D) was computed with *D* = (XH − XL)/[*n* (H − L)], where *XH* and *XL* are the sums of the station scores for the top and bottom 25% of candidates, respectively; moreover, n represents 25% of the total number of examinees (rounded up), and *H* and *L* represent the highest and the lowest attainable scores, respectively, on this question. The overall test difficulty was derived as *P* = Σ (*P*i × *k*i), where *P*i and *k*i represent the station difficulty and weight, respectively. Internal consistency reliability was measured with Cronbach’s α (range: 0–1), where larger values denote greater reliability. Interrater consistency was estimated via Pearson correlation analysis between paired examiner scores. Construct validity was inferred from the intercorrelations among the station scores. Content validity was evaluated by mapping every station to the core competencies mandated by the Beijing SRT guidelines.

### Statistical analysis

SPSS 27.0 software was used in this study. Normally distributed data are represented as the mean ± standard deviation $\left(\bar{x}\pm {{{\rm{s}}}}\right)$, and nonnormally distributed data are expressed as medians with interquartile ranges [Md (P25, P75)]. Pearson correlation was used for the normally distributed data; additionally, Kendall’s tau correlation was employed when normality assumptions were violated.

### Ethical considerations

This study protocol was approved by the Ethics Committee of Peking University First Hospital (No 2025R0307). Written informed consent was waived by the Institutional Review Board due to the retrospective nature of the study.

## Results

### Station design

The OSCE comprises six stations (Table 1). Beginning in 2021, the single case-analysis station was split into dedicated PET/CT and SPECT/CT stations, thus increasing the combined weight of image interpretation from 25% to 40%. To balance the overall examination time, the communication station was merged with the patient-interview station. The total OSCE time was increased from 120 to 130 min (Table 1).

### Number of candidates, pass rates and scores

The annual numbers of candidates were 14 (2019), 21 (2020), 27 (2021), 16 (2022), 14 (2023) and 21 (2024) for the years ranging from 2019 to 2024, respectively. The pass rate was determined to be 100% in all years, except for 2021 (96.3%, 26/27).

**Fig. 1:**
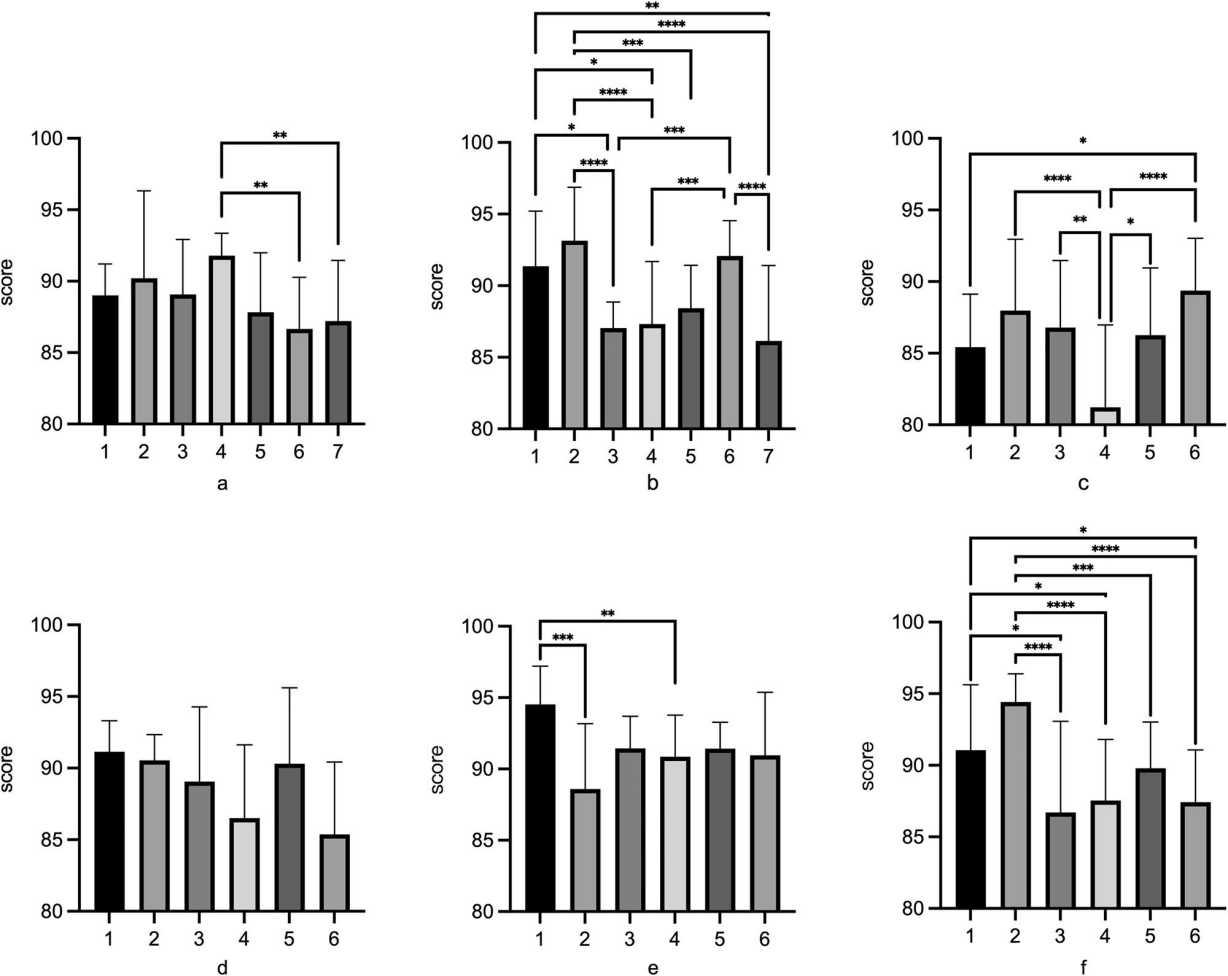
Scores of OSCE stations in each year, **a** (2019), **b** (2020), **c** (2021), **d** (2022), **e** (2023), **f** (2024). 1 high-activity room, 2 imaging room, 3 communication, 4 PET/CT case analysis, 5 SPECT/CT case analysis, 6 patient interview, 7 report writing (Fig a, b). 1 high-activity room, 2 imaging room, 3 PET/CT case analysis, 4 SPECT/C case analysis, 5 patient interview, 6 report writing (**c**–**f**). ^*^p < 0.05, ^**^p < 0.01, ^***^p < 0.001, ^****^p < 0.0001

The mean total scores significantly exceeded 85 points in every year, with significant between-station variation being observed (Fig. 1); moreover, the high-radioactivity room and imaging room operation stations frequently yielded higher marks relative to other stations.

### Difficulty and discrimination

**Fig. 2:**
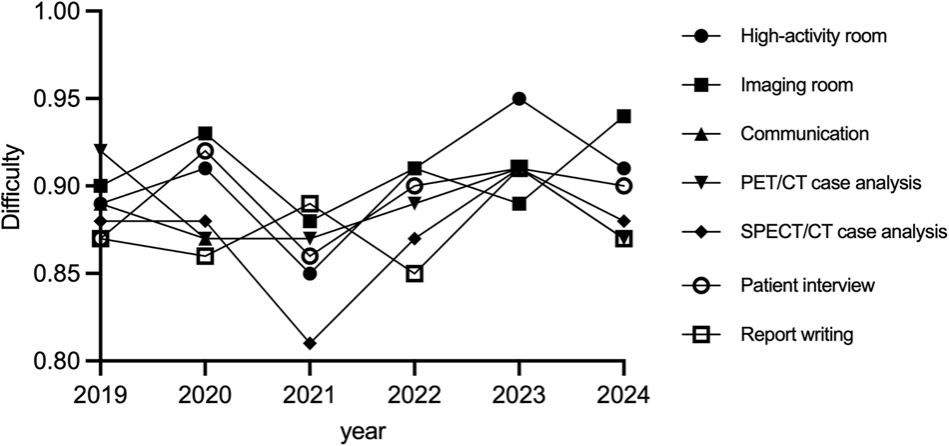
Difficulty of each OSCE station from 2019 to 2024

**Fig. 3:**
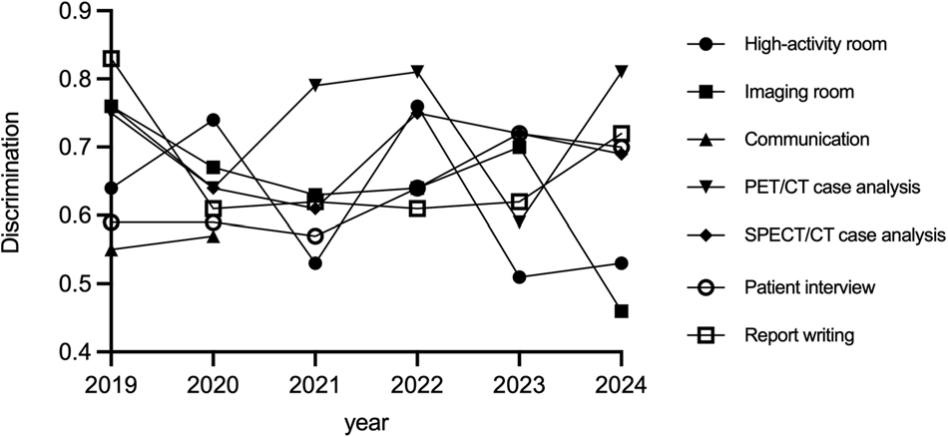
Discrimination of each OSCE station from 2019 to 2024

The station-level difficulty indices ranged from 0.81 to 0.95 across the six years, thereby yielding overall difficulties of 0.86–0.91 (Fig. 2). The station discrimination indices ranged from 0.46 to 0.83 (Fig. 3).

### Reliability

Among the 30 examiner pairs, 73.3% (22/30) demonstrated good interrater consistency (Table 2). The Cronbach’s α values were 0.66 (2019), 0.76 (2020), 0.57 (2021), 0.28 (2022), 0.05 (2023) and 0.69 (2024) for the years ranging from 2019 to 2024, respectively. The internal consistency reliability in 2022 and 2023 was observed to be relatively low.

**Table 2:** Interrater reliability of each OSCE station

Year	High-activity room		Imaging room		Communication		PET/CT case analysis		SPECT/CT case analysis		Patient interview	
	*r*	*p*	*r*	*p*	*r*	*p*	*r*	*p*	*r*	*p*	*r*	*p*
2019	0.54	0.046	0.93	0.00	0.66^a^	0.00	−0.29	0.31	0.31	0.28	0.21^a^	0.32
2020	0.27^a^	0.10	0.62^a^	0.00	0.06	0.79	0.77	0.00	0.24^a^	0.16	0.46	0.04
2021	0.45	0.00	0.51	0.00	/	/	0.73^a^	0.00	0.42^a^	0.00	0.43^a^	0.00
2022	0.19	0.48	0.47^a^	0.02	/	/	//	//	//	//	0.73	0.00
2023	0.49^a^	0.02	0.83	0.00	/	/	0.87	0.00	0.77	0.00	0.46	0.10
2024	0.87	0.00	0.41^a^	0.02	/	/	0.87^a^	0.00	0.75	0.00	0.86	0.00

*r* Correlation coefficient, *a* Kendall correlation coefficient; the remaining values are Pearson correlation coefficients, */* Communication was merged with the patient interview station, *//* only one examiner scoring was performed due to the COVID-19 pandemic

### Validity

The six-station OSCE (including high-radioactivity room operation, imaging room operation, PET/CT case analysis, SPECT/CT case analysis, patient interview and report writing) provides comprehensive coverage of foundational knowledge, clinical reasoning, practice and communication, thus satisfying the SRT requirements and confirming good content validity. The correlations among the various OSCE stations ranged from 0.31 to 0.72 (Tables 3 and 4), thus supporting moderate construct validity.

**Table 3:** OSCE structural validity (correlation of the stations) in 2019 and 2020

Year	Station	1	2	3	4	5	6	7
2019	1	1	0.72^**^	0.16	0.07	0.01	0.17	−0.24
	2	0.72^**^	1	0.23	0.32	0.05	0.49	0.14
	3	0.16	0.23	1	0.24	0.42	0.57^*^	0.16
	4	0.07	0.32	0.24	1	0.33	0.35	0.10
	5	0.01	0.05	0.42	0.33	1	0.49	0.05
	6	0.17	0.49	0.57^*^	0.35	0.49	1	0.37
	7	−0.24	0.14	0.16	0.10	0.05	0.37	1
2020	1	1.00	0.30	0.01	0.29	0.02	0.43^**^	0.19
	2	0.30	1.00	0.01	0.39^*^	0.23	0.15	0.43^**^
	3	0.01	0.01	1.00	−0.11	0.06	0.01	0.15
	4	0.29	0.39^*^	−0.11	1.00	0.35^*^	0.30	0.32
	5	0.02	0.23	0.06	0.35^*^	1.00	−0.09	0.42^*^
	6	0.43^**^	0.15	0.01	0.30	−0.09	1.00	0.06
	7	0.19	0.43^**^	0.15	0.32	0.42^*^	0.06	1.00

Note: 2019 shows the Pearson correlation coefficients, 2020 shows the Kendall correlation coefficients, ^**^p < 0.001, ^*^p < 0.05

1—high-activity room, 2—imaging room, 3—communication, 4—PET/CT case analysis, 5—SPECT/CT case analysis, 6—patient interview, 7—report writing

**Table 4:** OSCE structural validity (correlation of the stations) from 2021 to 2024

Year	Station	1	2	3	4	5	6
2021	1	1.00	−0.05	0.31^*^	0.12	0.15	−0.03
	2	−0.05	1.00	0.34^*^	−0.05	−0.04	−0.07
	3	0.31^*^	0.34^*^	1.00	0.306^*^	0.13	0.18
	4	0.12	−0.05	0.31^*^	1.00	0.07	0.45^**^
	5	0.15	−0.04	0.13	0.07	1.00	−0.11
	6	−0.03	−0.07	0.18	0.45^**^	−0.11	1.00
2022	1	1.00	0.05	0.03	−0.02	−0.09	−0.61^**^
	2	0.05	1.00	0.05	0.38	−0.02	−0.03
	3	0.03	0.05	1.00	0.20	0.37	−0.06
	4	−0.02	0.38	0.20	1.00	0.28	0.15
	5	−0.09	−0.02	0.37	0.28	1.00	−0.06
	6	−0.61^**^	−0.03	−0.06	0.15	−0.06	1.00
2023	1	1.00	0.22	0.17	0.28	−0.02	0.01
	2	0.22	1.00	0.15	0.28	−0.09	−0.26
	3	0.17	0.15	1.00	0.06	−0.09	−0.07
	4	0.28	0.28	0.06	1.00	0.16	−0.10
	5	−0.02	−0.09	−0.09	0.16	1.00	−0.07
	6	0.01	−0.26	−0.07	−0.10	−0.07	1.00
2024	1	1.00	0.32^*^	0.17	0.53^**^	0.20	0.34^*^
	2	0.32^*^	1.00	0.13	0.40^*^	0.29	0.14
	3	0.17	0.13	1.00	0.21	0.04	0.38*
	4	0.53^**^	0.40^*^	0.21	1.00	0.23	0.24
	5	0.20	0.29	0.04	0.23	1.00	0.10
	6	0.34^*^	0.14	0.38^*^	0.24	0.10	1.00

Kendall correlation coefficients, ^**^p < 0.001, ^*^p < 0.05

1—high-activity room, 2—imaging room, 3—PET/CT case analysis, 4—SPECT/CT case analysis, 5—patient interview, 6—report writing

### OSCE adaptations during COVID-19

Between 2020 and 2023, COVID-19-related questions were incorporated into the selected stations, with 6% of the content reported in 2020 declining to 4.2%, 1.65% and 1% in 2021, 2022 and 2023, respectively. In 2022, a remote OSCE was adopted.

## Discussion

This retrospective study analysed the OSCE data of the residents who participated in the examination for the certification of NM physicians from 2019 to 2024 in Beijing. We accurately evaluate the validity and reliability of the OSCE. The results demonstrated that the OSCE could effectively simulate clinical environments and provide a comprehensive assessment of the clinical competence of nuclear medicine (NM) residents.

### Analysis of OSCE effectiveness

The mean total scores exceeded 85 points, suggesting the residents generally met the competence thresholds specified in the SRT framework. Overall difficulty indices ranging from 0.86 to 0.91 were considered to be appropriately moderate for a criterion-referenced examination, wherein the primary goal is to confirm minimal competence (rather than to rank candidates). However, these consistently high scores and pass rates warrant critical security regarding examination rigour and discriminatory capacity. With the above overall difficulty indices, the examination appears overly lenient for a high-stakes certification assessment. Such high values may indicate potential ceiling effects, which may undermine the examination’s ability to detect subtle deficiencies or discriminate among levels of proficiency, thereby potentially compromising the rigour expected of a certification examination intended to protect public safety. Specifically, the two practical stations (high-radioactivity room and imaging room) frequently produced the highest scores (often observed at ≥ 90 points). While these tasks follow well-standardised protocols that facilitate precise quantitative evaluation, the resultant score clustering near the maximum suggests that current checklists may be overly prescriptive or insufficiently challenging. Therefore, while OSCE remains a valid assessment method for evaluating practical ability [11], the current implementation may not fully possess the necessary rigour for high-stakes decisions; future iterations should consider incorporating more demanding performance criteria to enhance discrimination and restore appropriate assessment rigour.

Our study revealed that the discrimination across the stations ranged between 0.46 and 0.83. Discrimination refers to the ability to differentiate examinees at different levels. Indices exceeding 0.4 are generally considered to be acceptable for certification examinations. Additionally, difficulty may influence discrimination. Excessively difficult or easy items typically exhibit poor discrimination, whereas moderately difficult items tend to demonstrate better discriminative power [12, 13]. Our findings confirm that the current OSCE blueprint adequately distinguishes between high- and low-performing residents, which is a result of the moderate difficulty levels that were observed.

Reliability is defined as the reproducibility of test results and refers to the consistency of scores that are obtained when the same examination is administered to the same group of examinees at different times or under various conditions. Reliability was assessed via internal consistency reliability and interrater reliability [14]. In our study, Cronbach’s α was used to evaluate internal consistency. A Cronbach’s α value greater than 0.7 is considered to be acceptable, whereas a value exceeding 0.8 indicates good internal consistency [8]. Chabrera et al analysed the OSCE scores of nursing students and reported an overall Cronbach’s α value as high as 0.84 [14]. In our study, the total Cronbach’s α value fluctuated between 0.05 and 0.76 across the years. Notably, values below the widely accepted threshold of 0.7 for high-stakes certification were observed, indicating suboptimal internal consistency reliability. The NM OSCE intentionally assesses distinct competence domains. Consequently, lower internal consistency reliability is expected compared with assessments of narrowly defined constructs. However, the reliability observed in 2022 (α = 0.28) and 2023 (α = 0.05) falls substantially below acceptable standards and is attributable to COVID-19 pandemic-related modifications. Moreover, the stations involving patient interviews, high-radioactivity rooms and imaging rooms were delivered remotely via prerecorded videos and evaluated by examiners online. Furthermore, case analysis was administered in written form. In addition, the examination was relatively easy, with a maximum mean score of 92.96 points being observed. These factors compressed score variance and attenuated the α value, especially in 2023. Overly simplistic items fail to discriminate candidates’ true abilities, thereby compromising the validity of the test. Overall, the results showed variability in internal consistency reliability. Cronbach’s α values fluctuate substantially, with extremely low values in 2022 and 2023. Future iterations should investigate the sources of the variability to ensure measurement stability. Interrater reliability remained satisfactory; specifically, 73% (22/30) of the paired-examiner stations achieved good agreement, which was facilitated by detailed scoring rubrics. The lower concordance observed at some of the stations reflected the use of substitute examiners (to avoid institutional conflicts of interest) and the unavoidable subjectivity inherent in certain items. Adherence to strict protocols and the limiting of examiner prompts may enhance reliability [15]. Additionally, an increase in the number of stations or the extension of the examination duration can also improve OSCE reliability. Peng et al reported that examinations with 5–10 stations (with each station occurring for no longer than 10 min) demonstrated better internal consistency reliability [16]. Although those methods allow for broader coverage of clinical knowledge and skills, along with improving the assessment of professional competence, logistical constraints (such as examiner fatigue, candidate burden and cost) must also be considered.

Content validity is ensured via the comprehensive mapping of every station to the CanMEDS-aligned SRT competences, including patient interviews, clinical reasoning, skill operations, auxiliary tests, medical documentation and communication. The interstation correlation range of 0.31–0.72 (Tables 3 and 4) indicates that our OSCE stations assess related yet distinct competence domains. As demonstrated by Varkey et al in their validation of an OSCE assessing practice-based learning and improvement and systems-based practice competencies, substantial variability in interstation correlations (range = 0.62–0.99) is theoretically expected and empirically observed in multi-domain assessment where stations intentionally assess distinct subcompetencies [17]. Our results demonstrate superior internal structure compared to their study, providing evidence of satisfactory internal structure validity.

Since the emergence of the COVID-19 pandemic in 2019, remote OSCEs have proliferated worldwide [7]. In our study, the 2022 remote OSCE (which was implemented in response to COVID-19) offered a glimpse of future possibilities. Although remote delivery may enhance accessibility and standardisation, the loss of real-time interaction can obscure nonverbal cues and emotional nuances [5]. Empirical comparisons with traditional face-to-face OSCEs are scarce; thus, international consensus on technical standards and quality assurance is urgently needed to facilitate the wider adoption of remote assessment formats.

### Identified issues and recommended solutions

The case analysis and report writing stations are the linchpins of the SRT. Residents were observed to underperform in case analysis in 2019, 2020, and 2024, thereby indicating weakness in integrative clinical reasoning, which is a trend that has also been reported in other fields such as nursing [14]. Residents must integrate clinical symptoms, laboratory data and images (such as CT, MRI, and ultrasound images) into a reasonable differential diagnosis. This ability can only be cultivated through proactive daily practice and reinforced by preceptors who continuously enhance their own clinical acumen and teaching strategies. The scores obtained at the SPECT/CT station were observed to be lower than those at the PET/CT station, which was most notably demonstrated in 2021. This result was caused by two factors. First, PET/CT examinations generate greater revenue. Second, the development and increasing application of positron-emitting probes have driven a progressive expansion in the variety and volume of PET/CT examinations, whereas SPECT/CT volumes have plateaued. Consequently, trainees receive comparatively limited practical exposure to SPECT/CT. Nevertheless, SPECT/CT remains clinically indispensable due to its cost-effectiveness and diagnostic specificity for particular indications. The preservation of high-quality SPECT/CT rotations is essential for maintaining balanced expertise.

The report writing station also yielded the lowest and most variable scores (as observed in 2019, 2020, and 2022). This station comprised two NM cases plus one ultrasonography case, and one CT case. This composition aligns with the resident program’s rotational training requirements: nine months in radiology and two months in ultrasound departments. Moreover, score loss mainly occurred in the non-NM components. This scenario highlights a training imbalance; specifically, residents value rotations in the NM and underestimate rotations in complementary imaging modalities. The mastery of CT and ultrasonography interpretation is indispensable for NM practice and should be completed in a serious manner.

The communication assessment was included in the patient-interview station with abbreviated timing, thereby leading to the risk of superficial evaluation. Communication skills are inherently subjective and best evaluated through structured interactions with SPs. The Kalamazoo Consensus identified the following seven essential sets of communication tasks: (1) building a doctor-patient relationship; (2) opening a discussion; (3) collecting information; (4) understanding the patient’s perspective; (5) sharing information; (6) reaching an agreement on problems and plans; and (7) providing closure [18]. Future OSCE blueprints should allocate rubric items aligned with these tasks to ensure rigorous assessment.

Workforce shortages in NM may lead to leniency bias in examiner scoring to compensate for the shortage of NM practitioners by allowing these NM candidates to pass the exam to as great of a degree as possible, which is reflected in the historically elevated pass rates in Beijing. Strict adherence to standardised benchmarks is critical for safeguarding practitioners’ competence. Interrater reliability can be improved by (i) allocating examiners who are free of institutional conflicts and (ii) including external examiners from outside of Beijing to enhance objectivity. Although these measures increase both the examination duration and cost, the investment is justified by the gains in reliability and public trust. Beginning in 2025, the report writing station employed dual-examiner scoring to attenuate individual rater bias. Finally, the current OSCE frameworks prioritise special knowledge and technical skills while providing limited attention to humanistic care, interprofessional collaboration and lifelong learning. The incorporation of validated tools that target these CanMEDS roles will broaden the scope of assessment and better prepare residents for clinical practice.

### Limitations

This study has several limitations. First, this work has a small sample size and high pass rates. The low annual candidate numbers and near-universal pass rates may limit discrimination in a high-stakes certification examination. Second, formal trainee feedback regarding the OSCE process was absent, which could provide valuable insights for protocol optimisation. As demonstrated by Alkhateeb et al [19], candidate feedback highlights the need for enhanced examiner training to improve fairness, as well as the importance of a comfortable waiting area and efficient station transitions to reduce examinee stress. Thirdly, a methodological constraint of this study was the reliance on a single-examiner evaluation in the report writing station due to resource constraints. This design precludes calculation of true inter-rater reliability and may introduce undetected rater bias. Finally, this study was limited by its single-centre, single-region design confined to Beijing. Consequently, the psychometric properties identified herein may need to be adapted when applied to other regions with distinct training frameworks or examinee demographics. Future research should employ multi-centre designs to validate these findings across diverse geographic and institutional contexts.

## Conclusion

In summary, the nuclear-medicine OSCE for Beijing residents has been successfully implemented. Station difficulty and discrimination remain within acceptable ranges for a criterion-referenced certification examination. Content validity is robust; specifically, the six stations collectively address the clinical skills, professional values, and clinical-reasoning domains specified in the CanMEDS framework and required by the SRT regulations. Moderate interstation correlations confirm both coherence and independence, thus supporting satisfactory construct validity. The evaluation of OSCE data from Beijing provides valuable insights for global OSCE research and is essential for informing the development of globally applicable OSCE guidelines in NM SRT.

## Data Availability

The datasets used in the current study are available from the corresponding author on reasonable request.

## Abbreviations

CanMEDS: Canadian Medical Education Directions for Specialists
NM: Nuclear medicine
OSCE: Objective structured clinical examination
PET/CT: Positron emission tomography/computed tomography
SP: Standardised patient
SPECT/CT: Single-photon emission computed tomography/computed tomograph
SRT: Standardized residency training
